# Genomic insights into the evolution and mechanisms of carbapenem-resistant hypervirulent *Klebsiella pneumoniae* co-harboring *bla*_KPC_ and *bla*_NDM_: implications for public health threat mitigation

**DOI:** 10.1186/s12941-024-00686-3

**Published:** 2024-03-29

**Authors:** Qian Wang, Yue Liu, Ran Chen, Meng Zhang, Zaifeng Si, Yueling Wang, Yan Jin, Yuanyuan Bai, Zhen Song, Xinglun Lu, Mingju Hao, Yingying Hao

**Affiliations:** 1 Department of Clinical Laboratory, Shandong Provincial Hospital Affiliated to Shandong First Medical University, Jinan, China; 2 Department of Clinical Laboratory, Shandong Provincial Hospital, Cheeloo College of Medicine, Shandong University, Jinan, Shandong China; 3 Shandong Medicine and Health Key Laboratory of Laboratory Medicine, Department of Clinical Laboratory Medicine, The First Affiliated Hospital of Shandong First Medical University & Shandong Provincial Qianfoshan Hospital, Jinan, China

**Keywords:** *Bla*_KPC_, *Bla*_NDM_, ST11-KL64, CR-HvKP, IncC-type plasmid

## Abstract

**Background:**

Carbapenem-resistant hypervirulent *Klebsiella pneumoniae* (CR-hvKP) co-producing *bla*_KPC_ and *bla*_NDM_ poses a serious threat to public health. This study aimed to investigate the mechanisms underlying the resistance and virulence of CR-hvKP isolates collected from a Chinese hospital, with a focus on *bla*_KPC_ and *bla*_NDM_ dual-positive hvKP strains.

**Methods:**

Five CR-hvKP strains were isolated from a teaching hospital in China. Antimicrobial susceptibility and plasmid stability testing, plasmid conjugation, pulsed-field gel electrophoresis, and whole-genome sequencing (WGS) were performed to examine the mechanisms of resistance and virulence. The virulence of CR-hvKP was evaluated through serum-killing assay and *Galleria mellonella* lethality experiments. Phylogenetic analysis based on 16 highly homologous carbapenem-resistant *K. pneumoniae* (CRKP) producing KPC-2 isolates from the same hospital was conducted to elucidate the potential evolutionary pathway of CRKP co-producing NDM and KPC.

**Results:**

WGS revealed that five isolates individually carried three unique plasmids: an IncFIB/IncHI1B-type virulence plasmid, IncFII/IncR-type plasmid harboring KPC-2 and IncC-type plasmid harboring NDM-1. The conjugation test results indicated that the transference of KPC-2 harboring IncFII/IncR-type plasmid was unsuccessful on their own, but could be transferred by forming a hybrid plasmid with the IncC plasmid harboring NDM. Further genetic analysis confirmed that the pJNKPN26-KPC plasmid was entirely integrated into the IncC-type plasmid via the copy-in route, which was mediated by Tn*As1* and IS*26*.

**Conclusion:**

KPC-NDM-CR-hvKP likely evolved from a KPC-2-CRKP ancestor and later acquired a highly transferable *bla*_NDM-1_ plasmid. ST11-KL64 CRKP exhibited enhanced plasticity. The identification of KPC-2-NDM-1-CR-hvKP highlights the urgent need for effective preventive strategies against aggravated accumulation of resistance genes.

**Supplementary Information:**

The online version contains supplementary material available at 10.1186/s12941-024-00686-3.

## Introduction

*Klebsiella pneumoniae* is known to cause both community-acquired and nosocomial infections globally and is regarded as one of the most prevalent and clinically relevant opportunistic pathogens [1]. With limited treatment options, carbapenem-resistant *K. pneumoniae* (CRKP) poses an enormous public health threat owing to increasing antibiotic resistance as a result of the acquisition of multiple mobile genetic elements [2].

Since 2017, China has experienced a concerning outbreak of ST11 carbapenem-resistant hypervirulent *K. pneumoniae* (ST11-CR-hvKP), associated with high mortality rates [3]. A nationwide survey found ST11-KL64 *K. pneumoniae* as the most prevalent CRKP clone in China, with *bla*_KPC-2_, *bla*_NDM-1_, and *bla*_OXA-48_ being the predominant carbapenemase genes among ST11-CRKP [4]. The emergence of the ST11-KL64 CR-hvKP clone in China is primarily attributable to the continuous coevolution of plasmids carrying hypervirulence and carbapenem resistance genes. These variants potentially develop in two directions: (i) CR-hvKP, an hvKP that acquires a plasmid encoding carbapenemase, and (ii) hypervirulent carbapenem-resistant *K. pneumoniae* (hv-CRKP), a CRKP strain that acquires a virulence plasmid. Initially, pLVPK-like virulence plasmids were described as nonconjugative; however, recent studies revealed that pLVPK-like virulence plasmids could be transferred from hvKP strains to ST11-CRKP with the help of a self-transferable IncF plasmid in various modes [5].

Of note, ST11-CR-hvKP co-harboring *bla*_KPC_ or *bla*_NDM_ has been increasingly reported, thereby necessitating urgent control measures. Nonetheless, evolutionary and dissemination strategies remain to be elucidated. A previous study based on the phylogenetic analysis of genomes from public databases showed that the formation of the superbug co-carrying NDM and KPC mainly emerged from a KPC-2-CRKP progenitor, which later acquired another highly transferable *bla*_NDM-1_ plasmid [2]. However, further genomic studies and experiments are required to verify this hypothesis. The present study aimed to investigate the mechanisms underlying the resistance and virulence of CR-hvKP isolates collected from a Chinese hospital, with a focus on *bla*_KPC_ and *bla*_NDM_ dual-positive hvKP strains.

## Materials and methods

### Bacterial strains

Five non-duplicate ST11-KL64 CR-KP isolates co-producing *bla*_KPC_ and *bla*_NDM_ were collected from Shandong Provincial Hospital between January 2017 and June 2020. Permission to report cases was obtained from patients or their representatives.

### Antimicrobial susceptibility testing

Antimicrobial susceptibility testing was conducted using the VITEK-2 Compact system (bioMérieux, France) in accordance with the Clinical and Laboratory Standards Institute guidelines. The minimum inhibitory concentrations (MICs) of tigecycline and colistin were determined using the broth dilution method and interpreted in accordance with the US Food and Drug Administration standards and the European Committee on Antimicrobial Susceptibility Testing (http://www.eucast.org/clinical_breakpoints) guidelines, respectively.

### Conjugation assay

The transfer ability of KPC and NDM harboring plasmids was assessed using JNKPN26 as the representative donor and *Escherichia coli* J53^AziR^ as the recipient strain. A second round of conjugation assay was conducted using transconjugants as donor strains and EC600 as the recipient strain to test the mobilization of the hybrid plasmids. Conjugation was performed in liquid Luria–Bertani (LB) medium incubated at 37 °C without shaking. The *bla*_KPC-2_- and/or *bla*_NDM-1_-bearing transconjugants were selected on China Blue lactose agar supplemented with sodium azide and ceftazidime. In order to improve the screening efficiency of transconjugants carrying KPC, the *bla*_NDM_ expression was suppressed by adding 0.1 mM EDTA to the screening plate. For all transconjugants, polymerase chain reaction (PCR) detection combined with XbaI and S1 pulsed-field gel electrophoresis (S1-PFGE) was employed to validate the presence of *bla*_NDM-1_ and/or *bla*_KPC-2_ harboring the plasmids. The conjugation frequency was determined by calculating the ratio of transconjugants to recipients based on the colony forming unit (CFU) count on serial dilution plates with matching antibiotics.

### Plasmid stability assay

Hybrid plasmid stability was assessed as previously described, with moderate modifications [2]. Briefly, isolates were cultured in LB broth at 37 °C with shaking (200 rpm) and serially passaged for 7 days (approximately 140 generations) with 1:1000 dilutions in antibiotic-free LB broth. Cultures were serially diluted and plated on Mueller Hinton agar plates without antibiotics every 24 h for 7 days. Two pairs of specific primers flanking the fusion site were designed to validate the fusion state of plasmids carrying *bla*_NDM-1_ and *bla*_KPC-2_. Furthermore, 48 bacterial colonies were randomly selected, and PCR was performed every 24 h. The primers used in the assay are listed in Additional file 3: Table S1.

### Whole-genome sequencing (WGS) and bioinformatics analysis

Genomic DNA sequencing was conducted by Novogene Co., Ltd. (Beijing, China) using both Illumina HiSeq and Nanopore platforms. Sequence assembly and annotation were conducted following the methodology outlined in previous research [6]. Samtools was used to assess the coverage [7].

Orthologous gene groups were identified using OrthoFinder version 2.3.12 [8]. Multiple sequence alignment was performed using MAFFT [9], and phylogenetic reconstruction was conducted using Gubbins [10]. Based on the sample separation time, a sample time-evolution relationship was constructed using BactDating [11]. Transmission tree inference was conducted using the Bayesian program TransPhylo [12].

### *Galleria mellonella* infection model

The virulence of CRKPs was examined using a *G. mellonella* infection model. Larvae weighing approximately 300 mg (Tianjin Huiyude Biotech Company, Tianjin, China) were kept in the dark at approximately 10 °C for backup. Overnight cultures of *K. pneumoniae* strains were washed with phosphate-buffered saline and further adjusted to concentrations of 1 × 10^5^, 1 × 10^6^, and 1 × 10^7^ CFU/mL. ATCC13883 and NTUH-K2044 were used as low- and high-virulence controls, respectively. Each group comprised eight *G. mellonella* larvae. The survival rate of *G. mellonella* was recorded every 12 h, and all experiments were performed in triplicate. The results for the *G. mellonella* model were analyzed using Kaplan–Meier survival curves and log-rank tests.

### Serum-killing assay

In vitro virulence was evaluated using a serum-killing assay, as described previously [13]. Briefly, 25 µL of bacterial suspension (at a concentration of 1 × 10^6^ CFU/mL) was added to 75 µL of mixed serum from 10 healthy humans for co-culture in a microtiter plate. After 0, 1, 2, and 3 h, the plates were inoculated with the mixture, and the number of viable bacteria was determined. The test was independently performed at least three times, and the percentage of CFU counts was characterized as the result of serum resistance, which was graded from 1 to 6. With normal human serum, a strain was generally considered to be resistant if it could attain grades 5–6, as previously described [13]. Unpaired two-sided Student’s t-test was performed for the strains, and data were presented as means ± standard deviation (SD).

## Results

### Clinical and strain features

**Fig. 1:**
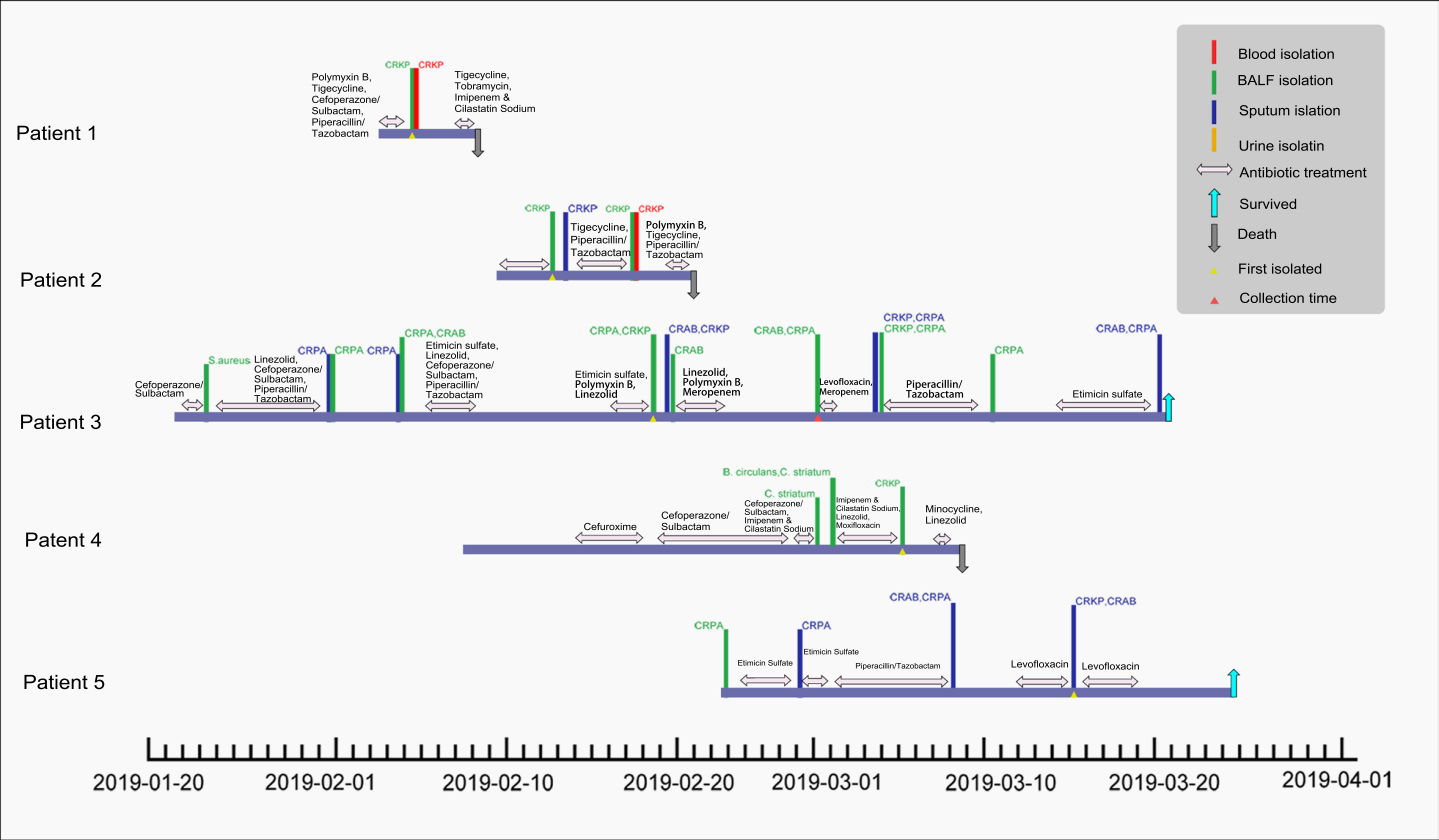
Epidemiology of *Klebsiella pneumoniae* outbreak cases. Colored text and bars represent the source from which the bacterial species were isolated, whereas colored triangles represent the time at which the bacterial species were isolated. CRKP: carbapenem-resistant *Klebsiella pneumoniae*; CRPA: carbapenem-resistant *Pseudomonas aeruginosa*; CRAB: carbapenem-resistant *Acinetobacter baumannii*; *S. aureus*: *Staphylococcus aureus*; *E. coli*: *Escherichia coli*; *B. circulans*: *Bacillus circulans*; *C. striatum*: *Corynebacterium striatum*

Patients with KPC-NDM-CR-KP infections were admitted to Shandong Provincial Hospital’s ICU from January to April 2019. Most had severe pneumonia, and one had postoperative pulmonary infection. Patients developed symptoms like pulmonary edema, pleural effusion, sputum, and shortness of breath, requiring mechanical ventilation. The strains were isolated within 3–42 days of admission. Patients 1 and 2 died due to severe pneumonia, sepsis, and multi-organ failure, while patient 4 discontinued treatment after septic shock. The other three improved with antibiotic therapy (Table 1, Fig. 1). All strains were highly resistant to β-lactam antibiotics, including carbapenems, but susceptible to tigecycline. JNKPN26 isolated from patient 3 was resistant to polymyxin (MIC > 64 mg/L) (Table 2).

**Table 1:** Clinical characteristics of patients with KPC-2-NDM-1-CR-hvKP

Variables/patients	Patient 1	Patient 2	Patient 3	Patient 4	Patient 5
Strain	JNKPN23	JNKPN24	JNKPN26	JNKPN29	JNKPN31
Clinical characteristics					
Age	70	70	44	56	69
Gender	Female	Female	Female	Female	Female
City	JINAN	JINAN	JINAN	JINAN	JINAN
Ward	ICU	ICU	ICU	ICU	ICU
Underlying conditions	Right oophorectomy, myocardial ischemia, chronic ischemic heart disease, respiratory failure	Hypertension, diabetes, dyslipidemia	Hepatic cyst, splenic cyst	Esophageal squamous cell carcinoma	Hypertension, diabetes, UTI
Invasive procedures					
Mechanical ventilation	Yes	Yes	Yes	Yes	Yes
Drainage catheters	Yes	Yes	Yes	Yes	Yes
Surgery	Yes	Yes	Yes	Yes	Yes
Date of specimen collection	2019/2/5	2019/2/15	2019/2/28	2019/3/6	2019/3/15
Infection type	Severe pneumonia, sepsis	Severe pneumonia, sepsis	Severe pneumonia	Severe pneumonia	Severe pneumonia
Prior antibiotic usage within 30 days	Yes	Yes	Yes	Yes	Yes
Empirical antimicrobial usage	CSL + POL + TZP + TGC	TZP + TGC	CSL + TZP + CIP + LZD + POL + ETS	CXM + CSL + ETS + LZD + MIN	ETS + TZP + LEV
Clinical presentations					
Temperature (max °C)	39.2 °C	38.6 °C	39.5 °C	39.1 °C	38.2 °C
WBC (×109/L)	27.83	29.49	28.73	20.93	16.04
Therapeutic antimicrobial usage	TOB + IPM + TGC	TZP + TGC + POL	LZD + POL + MEM + LEV + TZP + ETS	LZD + MIN	LEV
Clinical outcomes					
Days of mechanical ventilation	6	12	51	10	7
Duration of ICU stay (days)	6	12	56	29	30
Outcome	Death	Death	Survived	Death	Survived

*CSL* cefoperazone/sulbactam, *POL* polymyxin, *TZP* piperacillin/tazobactam, *IPM/CS* imipenem/cilastatin, *TOB* tobramycin, *TGC* tigecycline, *LZD* linezolid, *CIP* ciprofloxacin, *MEM* meropenem, *LEV* levofloxacin, *CXM* cefuroxime sodium, *MIN* minocycline, *ETS* etimicin sulfate, *UTI* urinary tract infection

**Table 2:** Microbiological characteristics of clinical isolated

Isolate	Carbapenemase	MLST	MIC (μg/mL)													
			TZP	CAZ	CRO	FEP	ATM	ETP	IPM	AMK	GEN	CIP	LVX	SXT	POL	TGC
JNKPN01	KPC-2	ST11	≥ 128	≥ 64	≥ 64	≥ 64	≥ 64	≥ 32	≥ 16	≥ 64	≥ 16	≥ 4	≥ 8	≤ 1	1	0.5
JNKPN03	KPC-2	ST11	≥ 128	≥ 64	≥ 64	≥ 64	≥ 64	≥ 32	≥ 16	≥ 64	≥ 16	≥ 4	≥ 8	≤ 1	0.5	2
JNKPN05	KPC-2	ST11	≥ 128	≥ 64	≥ 64	≥ 64	≥ 64	≥ 32	≥ 16	≤ 2	≥ 16	≥ 4	≥ 8	≤ 1	1	1
JNKPN15	KPC-2	ST11	≥ 128	≥ 64	≥ 64	≥ 64	≥ 64	≥ 32	≥ 16	≥ 64	≥ 16	≥ 4	≥ 8	≤ 1	1	0.5
JNKPN23	KPC-2; NDM-1	ST11	≥ 128	≥ 64	≥ 64	≥ 64	≥ 64	≥ 32	≥ 16	≥ 64	≥ 16	≥ 4	≥ 8	≥ 16	1	0.5
JNKPN24	KPC-2; NDM-1	ST11	≥ 128	≥ 64	≥ 64	≥ 64	≥ 64	≥ 32	≥ 16	≥ 64	≥ 16	≥ 4	≥ 8	≥ 16	0.5	1
JNKPN26	KPC-2; NDM-1	ST11	≥ 128	≥ 64	≥ 64	≥ 64	≥ 64	≥ 32	≥ 16	≥ 64	≥ 16	≥ 4	≥ 8	≥ 16	> 64	1
JNKPN28	KPC-2	ST11	≥ 128	≥ 64	≥ 64	≥ 64	≥ 64	≥ 32	≥ 16	≥ 64	≥ 16	≥ 4	≥ 8	≤ 1	0.5	0.5
JNKPN29	KPC-2; NDM-1	ST11	≥ 128	≥ 64	≥ 64	≥ 64	≥ 64	≥ 32	≥ 16	≥ 64	≥ 16	≥ 4	≥ 8	≥ 16	0.5	1
JNKPN30	NDM-1	ST11	≥ 128	≥ 64	≥ 64	≥ 64	≥ 64	≥ 32	≥ 16	≥ 64	≥ 16	≥ 4	≥ 8	≥ 16	0.5	2
JNKPN31	KPC-2; NDM-1	ST11	≥ 128	≥ 64	≥ 64	≥ 64	≥ 64	≥ 32	≥ 16	≥ 64	≥ 16	≥ 4	≥ 8	≥ 16	1	1
JNKPN34	KPC-2	ST11	≥ 128	≥ 64	≥ 64	≥ 64	≥ 64	≥ 32	≥ 16	≥ 64	≥ 16	≥ 4	≥ 8	≤ 1	1	1
JNKPN37	KPC-2	ST11	≥ 128	≥ 64	≥ 64	≥ 64	≥ 64	≥ 32	≥ 16	≥ 64	≥ 16	≥ 4	≥ 8	≤ 1	1	2
JNKPN38	KPC-2	ST11	≥ 128	≥ 64	≥ 64	≥ 64	≥ 64	≥ 32	≥ 16	≥ 64	≥ 16	≥ 4	≥ 8	≤ 1	0.5	0.75
JNKPN49	KPC-2	ST11	≥ 128	≥ 64	≥ 64	≥ 64	≥ 64	≥ 32	≥ 16	≥ 64	≥ 16	≥ 4	≥ 8	≥ 16	0.5	0.25
JNKPN58	KPC-2	ST11	≥ 128	≥ 64	≥ 64	≥ 64	≥ 64	≥ 32	≥ 16	≤ 2	≤ 1	≥ 4	≥ 8	≤ 1	0.5	1

*MIC* minimal inhibitory concentrations, *CRO* ceftriaxone, *FEP* cefepime, *CAZ* ceftazidime, *ATM* aztreonam, *TZP* piperacillin–tazobactam, *ETP* ertapenem, *IMP* imipenem, *SXT* trimethoprim–sulfamethoxazole, *AMK* amikacin, *GEN* gentamicin, *CIP* ciprofloxacin, *LVX* levofloxacin, *TGC* tigecycline, *POL* polymyxin

**Fig. 2:**
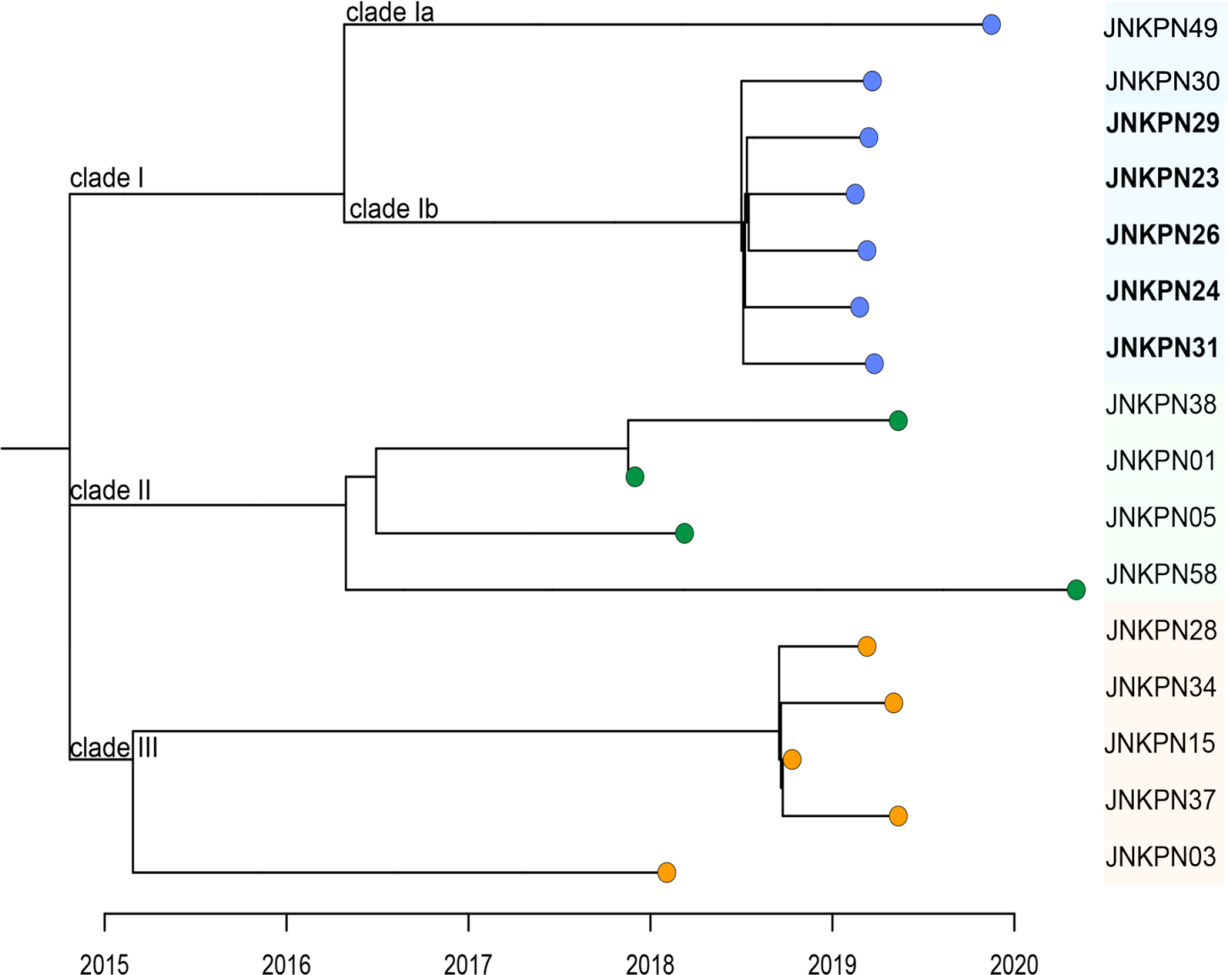
Hospital outbreak evolution analysis of CO-NDM-KPC-CRKP. The simulated outbreak evolution analysis of CRKP strains isolated in one health set. A phylogenetic tree was constructed using the sample separation time to infer evolutionary relationships among clinical isolates (BioProject PRJNA792451). The features of CO-NDM-KPC-CRKP associated with our hospital outbreak are highlighted in bold

**Fig. 3:**
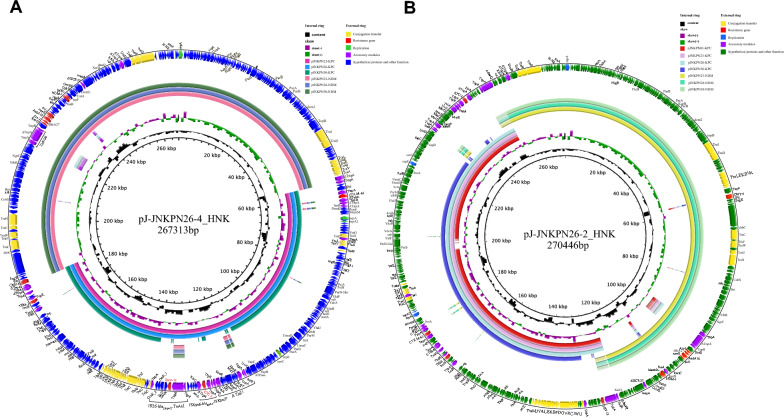
Comparative analysis of hybrid plasmids. Circular maps and alignments of pJ-JNKPN26-2_HNK (GenBank accession no. OR041627) and pJ-JNKPN26-4_HNK (GenBank accession no. OR041629) plasmids, showing their structural features and comparative analysis with other *bla*_*KPC*_ and *bla*_*NDM*_ harboring plasmids sequenced in this study

Phylogenetic analysis of these KPs and eleven other CRKPs from the hospital showed that five KPC-NDM-CR-KPs and NDM-CR-KP JNKPN30 formed a separate clade (Subclade Ib of Clade I) (Fig. 2). Six strains had identical PFGE profiles, indicating the same clone. This clade appeared to evolve between 2018 and 2019, possibly due to acquiring a *bla*_NDM_-positive plasmid. Genetic analysis of JNKPN23, JNKPN26 and JNKPN30 revealed a chromosome (~ 5470 kb) and three plasmids per isolate, revealing nearly 99.7% coverage more than 200× in depth: a virulence plasmid (~ 220 kb), a KPC-positive plasmid (~ 120 kb), and an NDM-positive plasmid (~ 140 kb), as confirmed by S1-PFGE. High similarity was observed among the KPC-encoding IncFII/IncR plasmid, which co-harboring *bla*_TEM-1_, *bla*_CTX-M-65_ and *bla*_SHV-12_ resistance genes, with more than 99% identity. The IncC plasmid carried *bla*_NDM_ and several AMR genes including *bla*_CMY-6_, *sul1*, *emrE*, *aadA16*, *dfrA27*, and *arr-3*, and exhibited a high similarity to others, with 100% coverage and 100% identity. JNKPN30 lacked *bla*_KPC_ gene, but an IncFII/IncR-type plasmid was identified within it. WGS showed the IncFII/IncR plasmid lost a 25-kb fragment (including *Δ*IS*Kpn6*-*bla*_KPC-2_-IS*Kpn27* and IS*26*-*bla*_SHV-12_-Tn*As1* resistance units, as well as *ΔTn21*) (Fig. 3). Notably, *mgrB* deletion was observed in the colistin-resistant strain JNKPN26 (Additional file 1: Fig. S1).

**Fig. 4:**
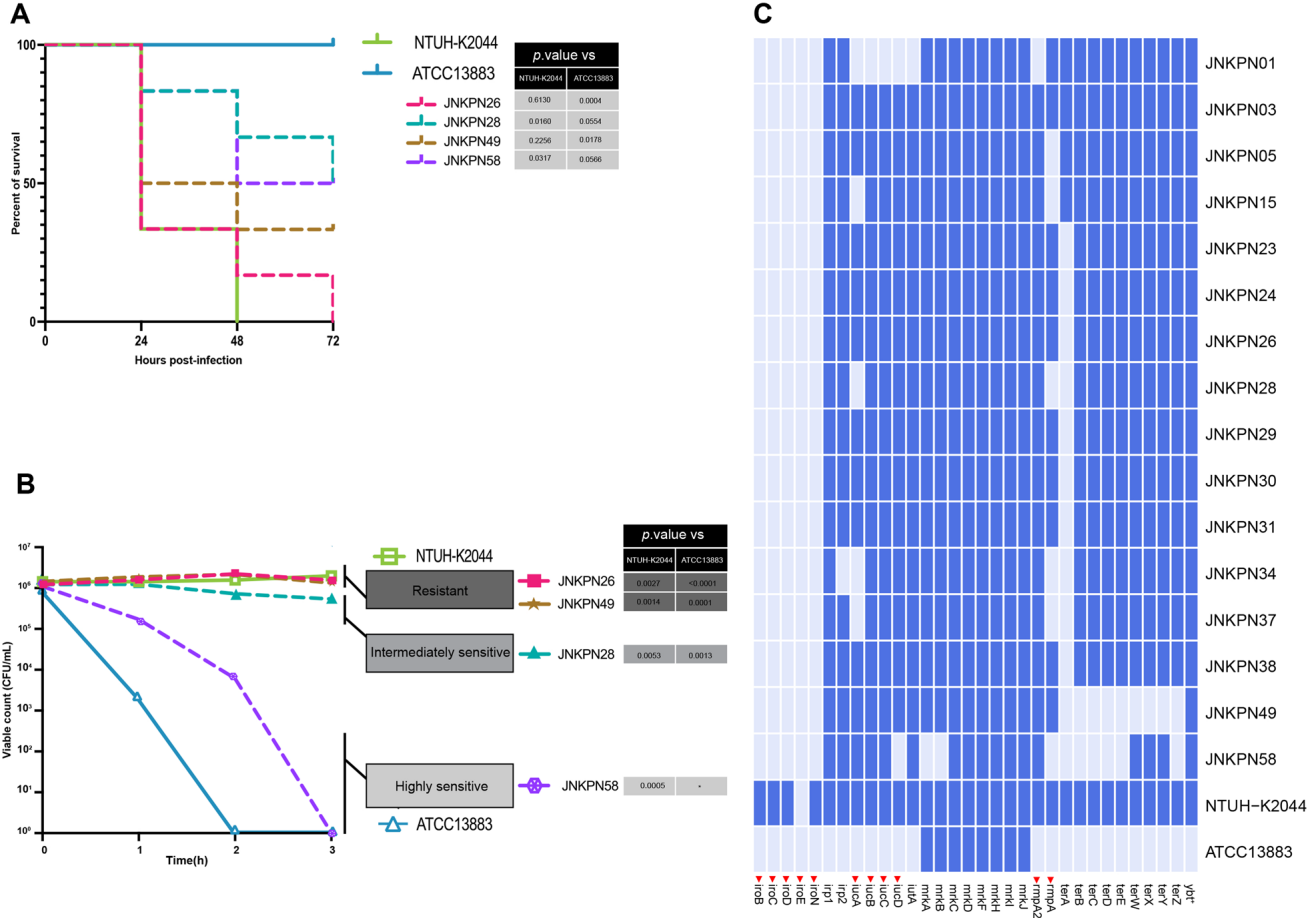
Analysis of the virulence and serum resistance of *Klebsiella pneumoniae* strains. **A** Survival rates of *Galleria mellonella* larvae injected with *Klebsiella pneumoniae* strains. Survival data were plotted using the Kaplan–Meier method, and the groups were compared using the log-rank test. Hypervirulent *K. pneumoniae* NTUH-K2044 and classic *K. pneumoniae* ATCC13883 strains were used as lower-virulence comparators. **B** Evaluation of the serum resistance of ST11 *Klebsiella pneumoniae* strains. Data are presented as mean (SD). The reference *K. pneumoniae* strain ATCC 13883 and the hypervirulent strain NTUH-K2044 were used as controls for the susceptible (ATCC 13883) and resistant (NTUH-K2044) grades, respectively. Significant differences (two-tailed unpaired t-test) in viable counts (3 h) of NTUH-K2044 and ATCC 13883 cells are shown in the table. *Unpaired t-test was not used because identical viable counts were recorded. **C** Virulence gene matrix of *Klebsiella pneumoniae* strains. The presence and absence of virulence genes are represented by dark blue and light blue boxes, respectively. Red triangles indicate virulence genes located in the plasmids. NTUH-K2044 and ATCC13883 are publicly available *K. pneumoniae* strains

All six strains harbored a 200–220-kb pLVPK-like virulence plasmid, identified as IncFIB/IncHI1B, carrying canonical virulence factors such as the regulator of the mucoid phenotype (*rmpA* and *rmpA2*), salmochelin (*iroBCDN*), aerobactin (*iucABCD* and *iutA*), and siderophore salmochelin (*iroBCDE* and *iroN*). The plasmid lacked antibiotic resistance genes and was devoid of T4SS, making it unable to undergo conjugative transfer. The *G. mellonella* virulence assay revealed that KPC-NDM-CR-KP JNKPN26 exhibited significantly higher virulence than ATCC13883 but showed similar virulence to the positive control NTUH-K2044. Furthermore, serum bactericidal assays indicated that KPC-NDM-CR-KP strains exhibited serum resistance, with a survival rate of approximately 78% after 60 min of incubation with pooled human serum. These phenotypic findings confirmed that KPC-NDM-CR-KP strains were hypervirulent (Fig. 4).

### Mobilization of the pJNKPN26-KPC plasmid with the help of the conjugative pJNKPN26-NDM plasmid

Conjugation assays demonstrated that the NDM-1-positive plasmid, pJNKPN26-NDM, could efficiently self-transfer to *E. coli* J53 with a high frequency of 3.56 × 10^–3^. In contrast, KPC-positive plasmid had a much lower transfer frequency of 1.8 × 10^–9^. Interestingly, all the KPC-positive transconjugants selected from plates containing EDTA, also carried the NDM bearing IncC plasmids (Table 3).

**Table 3:** Microbiological characteristics of transconjugants

Isolate	Carbapenemase	MIC (μg/mL)											
		CRO	CAZ	ATM	TZP	ETP	IMP	SXT	AK	GN	CIP	LEV	FOS
J-JNKPN23	NDM-1	≥ 64	≥ 64	4	≥ 128	≥ 8	≥ 16	≥ 16	≥ 64	≥ 16	≤ 0.25	≤ 0.25	≤ 32
J-JNKPN23-1	NDM-1, KPC-2	≥ 64	≥ 64	16	64	≥ 8	≥ 16	≥ 320	≥ 64	≥ 16	≤ 0.25	≤ 0.25	≤ 32
J-JNKPN24	NDM-1	≥ 64	≥ 64	4	≥ 128	≥ 8	≥ 16	≥ 16	≥ 64	≥ 16	≤ 0.25	≤ 0.25	≤ 32
J-JNKPN26-1	NDM-1, KPC-2	≥ 64	≥ 64	4	≥ 128	≥ 8	≥ 16	≥ 320	≥ 64	≥ 16	≤ 0.25	≤ 0.25	≤ 32
J-JNKPN26-2	NDM-1, KPC-2	≥ 64	≥ 64	4	≥ 128	≥ 8	≥ 16	≥ 320	≥ 64	≥ 16	≤ 0.25	≤ 0.25	≤ 32
J-JNKPN26-3	NDM-1, KPC-2	≥ 64	≥ 64	4	≥ 128	≥ 8	≥ 16	≥ 320	≥ 64	≥ 16	≤ 0.25	≤ 0.25	≤ 32
J-JNKPN26-4	NDM-1, KPC-2	≥ 64	≥ 64	4	≥ 128	≥ 8	≥ 16	≥ 320	≥ 64	≥ 16	≤ 0.25	≤ 0.25	≤ 32
J-JNKPN26-5	NDM-1	≥ 64	≥ 64	4	≥ 128	≥ 8	≥ 16	≥ 16	≥ 64	≥ 16	≤ 0.25	≤ 0.25	≤ 32
J-JNKPN29	NDM-1	≥ 64	≥ 64	2	≥ 128	≥ 8	≥ 16	≥ 16	≥ 64	≥ 16	≤ 0.25	≤ 0.25	≤ 32
J-JNKPN30	NDM-1	≥ 64	≥ 64	4	≥ 128	≥ 8	≥ 16	≥ 16	≥ 64	≥ 16	≤ 0.25	≤ 0.25	≤ 32
J-JNKPN31	NDM-1	≥ 64	≥ 64	≥ 64	≥ 128	≥ 8	≥ 16	≥ 16	≥ 64	≥ 16	≤ 0.25	≤ 0.25	≤ 32
J-JNKPN58	KPC-2	≥ 64	≥ 64	≥ 64	≥ 128	≥ 8	≥ 16	≤ 1	≤ 2	≤ 1	≤ 0.25	≤ 0.25	≤ 32

*MIC* minimal inhibitory concentrations, *CRO* ceftriaxone, *CAZ* ceftazidime, *ATM* aztreonam, *TZP* piperacillin–tazobactam, *ETP* ertapenem, *IMP* imipenem, *SXT* trimethoprim–sulfamethoxazole, *AMK* amikacin, *GEN* gentamicin, *CIP* ciprofloxacin, *LVX* levofloxacin

The IncC plasmid harbored a complete conjugative transfer-related module, including an oriT region, a relaxase of the MobH family, a type IV coupling protein (T4CP), and a *tra* gene cluster that coded for type IV T4SS. In contrast, relaxase and T4CP were absent in the pJNKPN26-KPC plasmid, suggesting that the KPC plasmid transfer relied on assistance from the IncC plasmid.

**Fig. 5:**
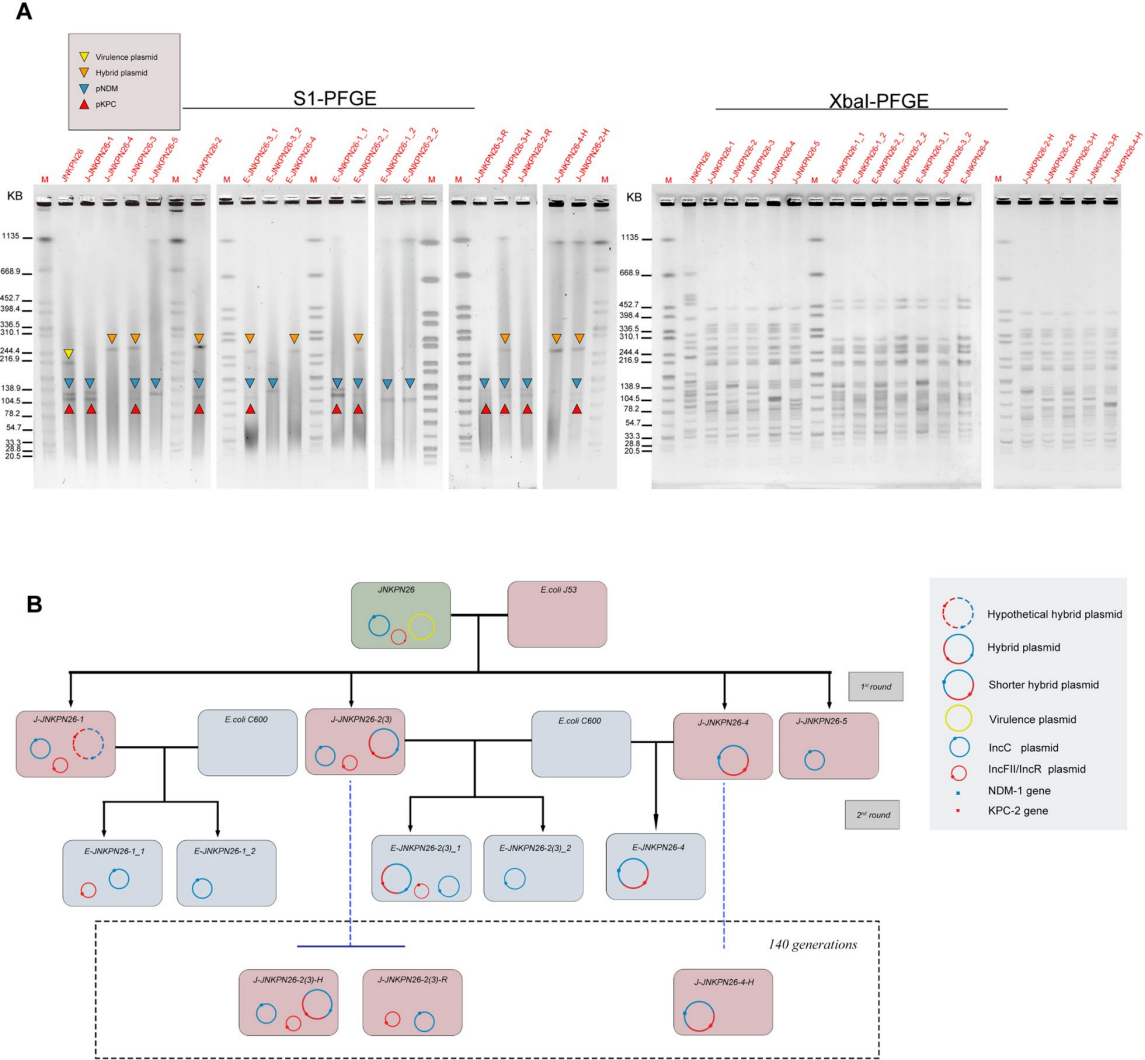
Mobilization of pJNKPN26-NDM and pJNKPN26-KPC. **A** XbaI and S1-PFGE of *K. pneumoniae* JNKPN26, as well as their corresponding transconjugants. Differently colored triangles denote different plasmids: virulence plasmid pJNKPN26-Vir (blue triangles), IncFII/IncR plasmid pJNKPN-KPC (yellow triangles), IncC plasmid pJNKPN-NDM (green triangles), and novel hybrid plasmid (orange triangles). **B** Schematic representation of a round of conjugation assays and the state of hybrid plasmid after passage. Rounded rectangles of the same color represent the same strains. Blue, red, and yellow circles denote pJNKPN26-NDM or its derivatives, pJNKPN26-KPC or its derivatives, and pJNKPN26-Vir, respectively

The plasmid state of transconjugants coharboring NDM and KPC was assessed with S1-PFGE. In addition to NDM and KPC located on separate plasmids, similar to the clinical strain (~ 140 kb and ~ 120 kb), two extra S1 patterns were detected in the conjugates. Briefly, the transconjugants co-harboring NDM and KPC displayed three different S1-PFGE profiles: (i) J-JNKPN26-1, which carried two independent plasmids (approximately 140 kb and 120 kb); (ii) J-JNKPN26-2 and J-JNKPN26-3, which contained a novel large plasmid (approximately 270 kb) and two smaller plasmids (approximately 140 kb and 120 kb, respectively); and (iii) J-JNKPN26-4, which possessed a novel large plasmid (approximately 270 kb) that was smaller than the novel plasmids found in J-JNKPN26-2 and J-JNKPN26-3 (Fig. 5, Additional file 2: Fig. S2).

**Fig. 6:**
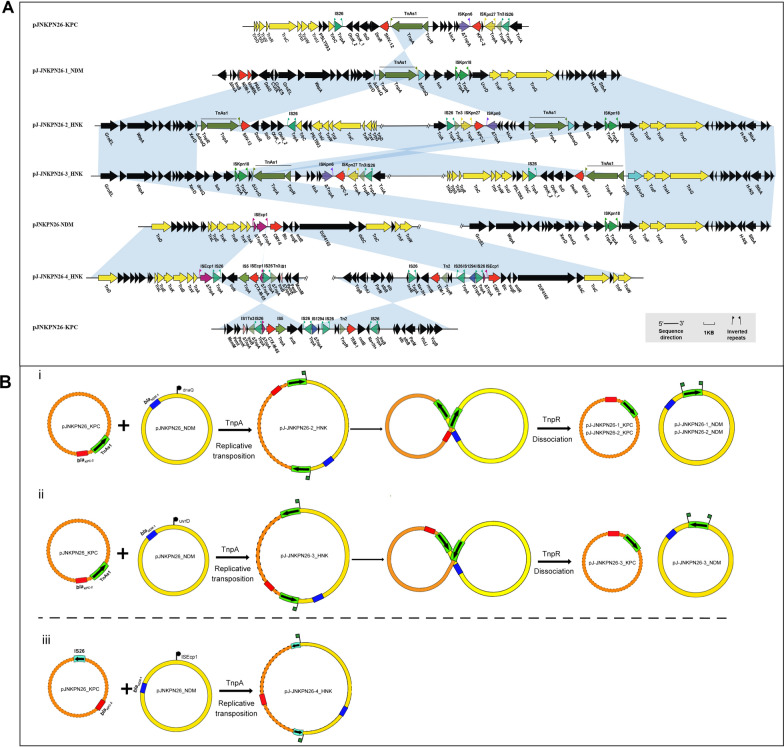
Genetic structures of the conjugative hybrid resistance plasmid fusion regions. **A** Alignment of hybrid resistance plasmids pJ-JNKPN26-1-NDM, pJ-JNKPN26-2_HNK, pJ-JNKPN26-3_HNK, and pJ-JNKPN26-4_HNK with parental plasmids from JNKPN26. Black, yellow, red, cyan-blue, and other colored arrows indicate other function protein, conjugation transfer, resistance gene, IS insertion site, and mobile element protein, respectively. The evidence for the occurrence of the fusion event in transconjugant J-JNKPN261-1 was the emergence of Tn*As1* on pJ-JNKPN26-1-NDM. **C** The resistance genes are indicated by rectangles. A target site and subsequent 8-bp duplications are indicated by a vertical flag. The relative frequencies of IS*26*- and Tn*As1*-mediated reactions are indicated by blue and green arrows, respectively. Proposed mechanisms of plasmid fusion. **i** pJ-JNKPN26-1_NDM and pJNKPN26-2_HNK: Tn*As1* attacked the *dnaQ* gene of pJNKPN26-NDM, leading to the formation of fusion plasmids through a replicative transposition mechanism. **ii** pJ-JNKPN26-3_HNK: Tn*As1* interrupted the *uvrD* gene of pJNKPN26-NDM, leading to the reverse insertion of pJNKPN26-KPC into pJ-JNKPN26-1-NDM forming plasmid pJ-JNKPN26-3_HNK. **iii** pJ-JNKPN26-4_HNK: formation of cointegration was mediated by IS*26* interrupting IS*Ecp1*

In pattern (i), pJ-JNKPN26-1_NDM and pJ-JNKPN26-1_KPC were almost identical with NDM and KPC harboring the plasmids in the clinical strain; nevertheless, inverted repeats (IRs) at the 5′-end repeat of Tn*As1* were only present within *dnaQ* in pJ-JNKPN26-1_NDM. Thus, the recombinant junction mediated by Tn*As1* was supposed to occur (Fig. 6).

In pattern (ii), three plasmids existed in the transconjugant J-JNKPN26-2—namely, pJ-JNKPN26-2_HNK (270,446 bp, GenBank accession number: OR041627), pJNKPN26-2_NDM (144,158 bp), and pJ-JNKPN26-2_KPC (126,207 bp). The transconjugant J-JNKPN26-3 harbored three plasmids—namely, pJ-JNKPN26-3_HNK (270,443 bp, GenBank accession number: OR041628), pJ-JNKPN26-3_NDM (144,239 bp) and pJ-JNKPN26-3_KPC (126,207 bp).

In pattern (iii), one large plasmid was identified in the transconjugant J-JNKPN26-4 with a size of 267,313 bp (Fig. 3). Comparative genetic analysis revealed that pJ-JNKPN26-4_HNK (GenBank accession number: OR041629) was a fusion plasmid formed from pJ-JNKPN26-KPC and pJNKPN26-NDM, mediated by the insertion sequence IS*26*. The two IS*26* sequences around the infusion site of IS*Ecp1* shared a 14-bp terminal IR sequence and 12-bp direct target repeats, suggesting that the recombinant junction was supposed to occur (Fig. 6).

Further genomic sequencing suggested that pJNKPN26-KPC underwent complete integration into pJNKPN26-NDM within the *dnaQ*, *uvrD*, and IS*Ecp1* genes in all three patterns. Additionally, genetic comparative analysis showed that Tn*As1* in the KPC-2 positive plasmid mediated the formation of pJ-JNKPN26-2_HNK and pJ-JNKPN26-3_HNK hybrid plasmids. The insertion sites in pJ-JNKPN26-2_HNK and pJ-JNKPN26-3_HNK were *dnaQ* and *uvrD*, respectively (Fig. 6). PCR targeting the region spanning the integration site of the fusion plasmid pJ-JNKPN26-2_HNK confirmed the hybrid state and fusion site. The Tn*As1*-mediated fusion event in pJ-JNKPN26-KPC targeting the *dnaQ* or *uvrD* gene of pJNKPN26-NDM was reversible.

### Stability and mobilization of hybrid plasmids

The stability of the hybrid plasmids pJ-JNKPN26-2_HNK, pJ-JNKPN26-3_HNK, and pJ-JNKPN26-4_HNK during passage was verified by conducting PCR on the fusion fragments flanking the susceptible recombination sites of the hybrid plasmids. The Tn*As1*-mediated heterozygous plasmids (pJ-JNKPN26-2_HNK and pJ-JNKPN26-3_HNK) showed poor stability in the heterozygous state on day 7 (stability of 54% and 57%, respectively). In contrast, the IS*26*-mediated fusion plasmid (pJ-JNKPN26-4-HNK) remained stable (stability of 100%) during passage for 7 days in an antibiotic-free environment, indicating that the plasmid had relatively high stability (Fig. 5). However, the KPC-2 and NDM-1 resistance genes could be detected by PCR from all transconjugate colonies after passage on day 7.

A second round of conjugation assay was performed to evaluate the transfer capability of hybrid plasmids. The results confirmed that all hybrid plasmids could be successfully transferred from the J53 transconjugant to the *E. coli* C600 recipient strain. The conjugative transfer efficiencies ranged from 4 × 10^–5^ to 8.69 × 10^–6^ and were lower than those of the exclusive IncC plasmid.

## Discussion

ST11-KL64 is an internationally distributed lineage of CRKP and represents the most common type in China, known for its abundance of virulence and resistance genes [14]. Rapid reshaping and diversification of the genomic pool of ST11-K64 CR-hvKP strains are facilitated by mobile genetic elements, even at a short time after the onset of the outbreak [15]. Previously reported strains with coexisting KPC and NDM were often sporadic [16]. As these strains have been increasingly reported worldwide, their stability, transmission capacity, and mechanism of action have drawn considerable attention [2, 17]. Recently, KPC-2-NDM-1-CRKP had been shown to be stable in the environment and could be nosocomially transmitted among patients [18]. Limited treatment choices, particularly for those carrying a typical hypervirulence plasmid, have led to a higher fatality rate in infected patients and have become a severe clinical challenge [2]. In this study, among six patients with nosocomial infections caused by KPC-2-NDM-1-CRKP, two died, whereas one discontinued treatment. All six strains harbored a 200–220-kb pLVPK-like virulence plasmid exhibiting a hypervirulent phenotype similar to the canonical hvKP NUTH-K2044 strain.

Colistin-based combination therapy is a commonly used last-resort agent for treating severe CRKP infections [19]. In this study, a colistin-resistant strain, JNKPN26, with a PFGE profile identical to that of other KPC-2-NDM-1-CRKPs, was isolated from patient 3. Comparative genetic analysis was conducted to identify the deletion of the *mgrB* region and its microevolution in vivo. We retrospectively analyzed the medical records of patient 3. After ten day’s high-dose colistin therapy, the colistin-resistant JNKPN26 strain was isolated from the patient’s sputum. In this case, colistin exposure was suspected to be an independent risk factor for the emergence of colistin resistance in vivo. These strains may have experienced rapid evolution and nosocomial outbreaks under antibiotic screening pressure.

Based on the retrospective genomic surveillance of CRKP strains at our hospital, our phylogenetic analysis suggested that the KPC-2-NDM-1-CRKP strains likely originated from the progenitor KPC-2-CRKP that acquired a highly transferable *bla*_NDM-1_ plasmid. This finding is consistent with previous studies that conducted genomic analyses using publicly available complete genome sequences [2].

We attempted to experimentally assess the stability and transmission of carbapenemase-carrying plasmids, specifically the pJNKPN26-KPC plasmid, but failed to obtain transconjugants with this plasmid alone. However, pJNKPN26-KPC could be transferred accompanied by the co-transference of *bla*_NDM_ harboring the IncC plasmid. According to a previous study, only 8.0% of IncFII-IncR KPC-2-encoding plasmids from CRKP can be transferred to *E. coli* [20]. In this study, analysis of pJNKPN26-KPC by oriTfinder revealed that the relaxase gene and T4CP were absent, establishing that the IncFII-IncR-type plasmid employed an inactive type IV secretion system (T4SS). Further S1-PFGE and WGS revealed that the pJNKPN26-KPC plasmid was transferred through complete integration into the self-transferable IncC-type plasmid pJNKPN26-NDM to form a novel hybrid plasmid. Cointegration formed by the copy-in route was mediated by Tn*As1* and IS*26*. Tn*As1* or IS*26* from pJNKPN26-KPC interrupted the IncC plasmid targeting at random sites, such as *dnaQ*, *uvrD*, and IS*Ecp1*, followed by a replication step that duplicated themselves. Interestingly, the hybrid resolved back into two individual plasmids during the recombination event mediated by Tn*As1*. These findings suggested that NDM harboring the plasmid acted as a helper plasmid for mobilization of the KPC-bearing plasmid.

We further examined the genetic architecture and hereditary patterns of the hybrid plasmids mediated by the two mobile elements. The first route was a combination mediated by IS*26* on pJNKPN26-KPC inserted into IS*Ecp1* on the NDM-bearing IncC-type plasmid pJNKPN26-NDM. Cointegration was experimentally determined to be stable without dissociation after 7 days of passage. The other route is mediated by Tn*As1* in pJNKPN26-KPC inserted into *dnaQ* or *uvrD* in pJNKPN26-NDM. In contrast to IS*26* mediated recombination, all three cointegrates mediated by Tn*As1* can be dissociated after transfer or passage, resulting in more flexibility during the shuttle process of the mobile element. IS*26* has been widely reported to play a critical role in the dissemination of antibiotic resistance genes in gram-negative bacteria, which have been shown to form cointegrates both by a copy-in mechanism involving one insertion sequence (IS) and by a targeted conservation mechanism involving two ISs [21]. In addition, the Tn*3* family is ubiquitous in bacteria, molding host genomes via a paste-and-copy mechanism [22]. It has rarely been reported that Tn*As1* carries the entire large plasmid and acts as a transposition module through the copy-in mode. Tn*As1* contains a transposase (*tnpA*) with an integration function, a resolvase (*tnpR*) with a dissociation function, and an *res* site containing both promoters and IRs at both ends. We speculated that *tnpR* in Tn*As1* mainly contributes to the dissociation of the integration intermediate. Among the co-integration events, Tn*As1* was detected at a higher frequency than IS*26*, suggesting that Tn*As1* may be a more active driver of translocatable units.

IncC plasmids have been widely studied because of their large size, conjugative transfer capability, pan-host prevalence worldwide, and ability to carry multiple drug resistance genes, especially CMY and *bla*_NDM_ [23]. NDM harboring IncC plasmids are widespread among a variety of Enterobacteriaceae strains in many countries [24]. IncC-type plasmids accounted for 52.4% of NDM-producing CRKP isolates in Pakistan [25]. In China, IncC-type plasmids are essential contributors to the dissemination of NDM, accounting for 12.28% of all *bla*_NDM_ harboring the plasmids in *K. pneumoniae* [26]. Recently, a *bla*_NDM-1_-bearing cointegrated plasmid from a clinical Salmonella Lomita strain was generated from IS 26 mediated integration of an IncX3 and IncC plasmid. In addition to co-transference by forming hybrid plasmids, insertion sequence-mediated rearrangements may promote the diversity and rapid evolution of the IncC genome [27]. Further studies should investigate the fitness cost of excellent compatibility and transferability after the insertion of a large sequence.

## Conclusions

In conclusion, the present study investigated the evolutionary pathway based on a phylogenetic analysis of a cluster of highly homologous CRKPs isolated from one health set. Our results verified that KPC-2-NDM-1-CRKP formed from a KPC-2-CRKP strain that acquired a highly transferable *bla*_*NDM-1*_ plasmid in silico. Unfortunately, not all the strains isolated from related patients and environment were collected and sequenced. It’s impossible to determine the origin of *bla*_*NDM*_ bearing plasmid in the real world. More well planed sample and sequencing are needed for further confirmation. Interestingly, KPC harboring the IncFII/IncR-type plasmid could be transferred by the IncC-type plasmid via the formation of a large hybrid plasmid mediated by IS*26* or Tn*As1*. A better understanding of the mechanisms underlying and triggering co-integration may facilitate the development of interventional measures to curb the formation and dissemination of such elements.

### Supplementary Information

**Additional file 1: Figure S1.** Comparative analysis of mgrB-related region. Alignment of mgrB-related region in colistin-resistant strain JNKPN26 with colistin-susceptible strain JNKPN30.

**Additional file 2: Figure S2.** Original PFGE Pattern.

**Additional file 3: Table S1.** The primers used in this study.

## Data Availability

Data will be made available on request.

## Abbreviations

CR-hvKP: Carbapenem-resistant hypervirulent *Klebsiella pneumoniae*
hv-CRKP: Hypervirulent carbapenem-resistant *K. pneumoniae*
CRKP: Carbapenem-resistant *K. pneumoniae*
WGS: Whole-genome sequencing
MICs: Minimum inhibitory concentrations
CFU: Colony forming unit
PCR: Polymerase chain reaction
T4SS: Type IV secretion system
ISs: Insertion sequences
IRs: Inverted repeats
PFGE: Pulsed-field gel electrophoresis
CZA: Ceftazidime/avibactam
