# Supplementary figures and images for: Genomic insights into the evolution and mechanisms of carbapenem-resistant hypervirulent Klebsiella pneumoniae co-harboring blaKPC and blaNDM: implications for public health threat mitigation
Source: Ann Clin Microbiol Antimicrob. 2024 Mar 29;23:27. doi: 10.1186/s12941-024-00686-3 (PMC10981300; doi:10.1186/s12941-024-00686-3)

JNKPN30-chromosome

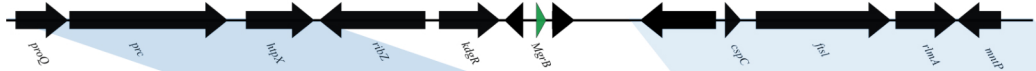

JNKPN26-chromosome

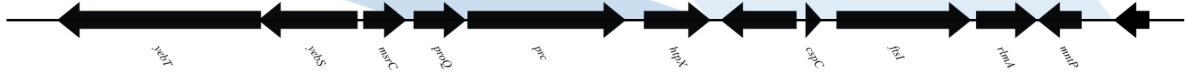

Supplement: Supplementary file 1 — Additional file 1: Figure S1. Comparative analysis of mgrB-related region. Alignment of mgrB-related region in colistin-resistant strain JNKPN26 with colistin-susceptible strain JNKPN30. [file 12941_2024_686_MOESM1_ESM.pdf]

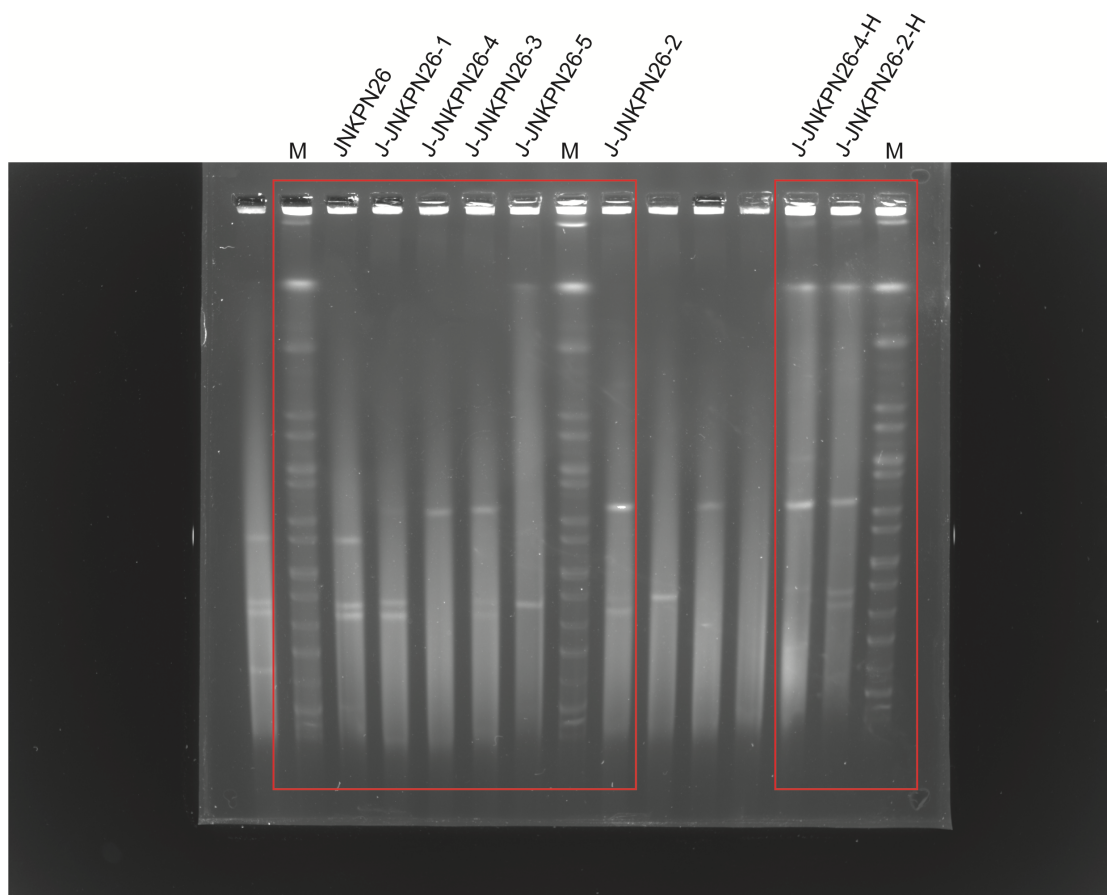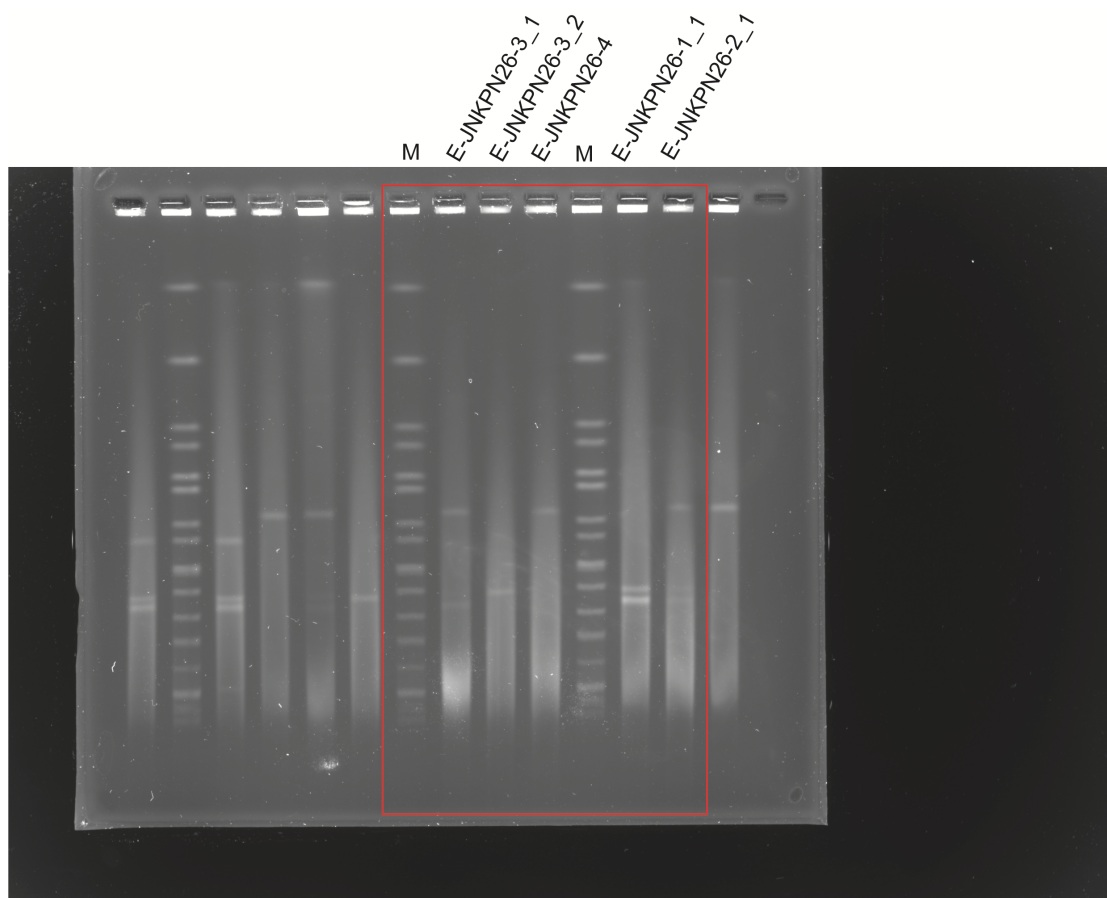

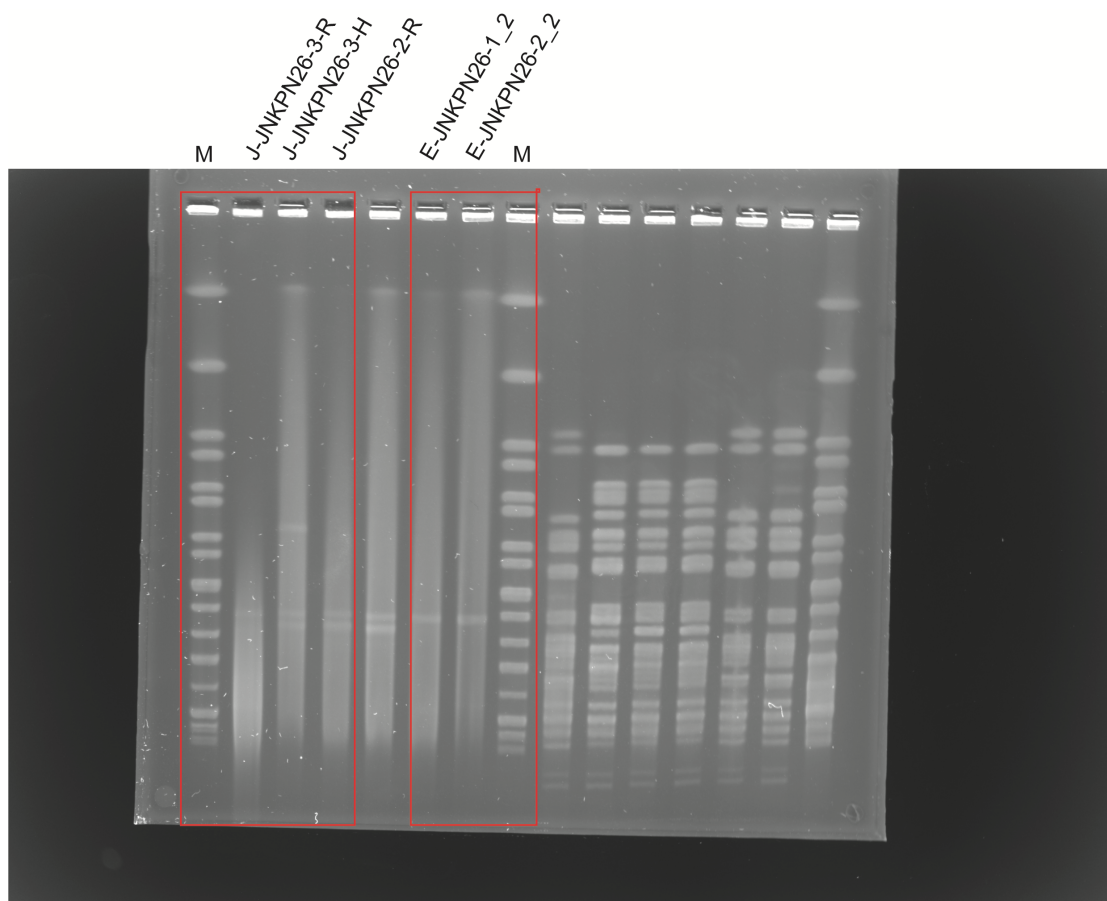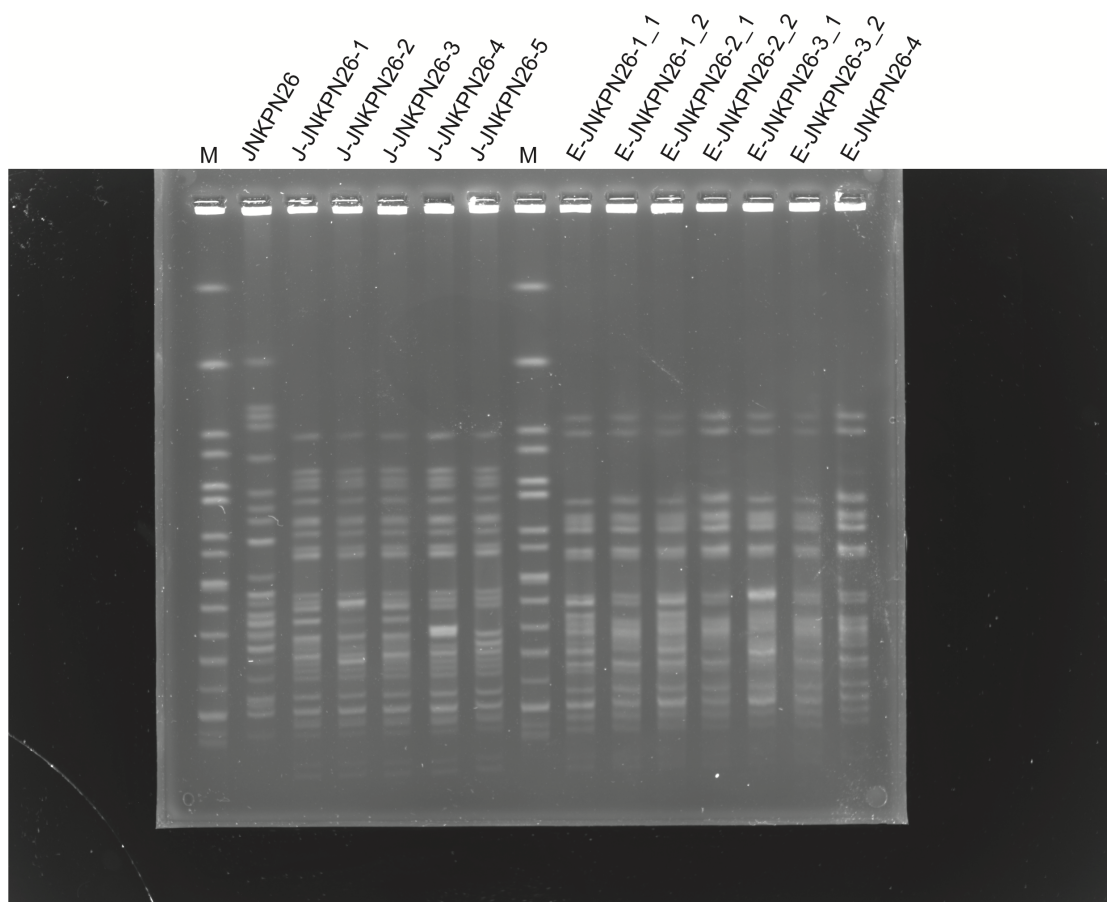

M JUNKPN26-2-H  
JUNKPN26-2-R JUNKPN26-3-H  
JUNKPN26-3-R JUNKPN26-4-H

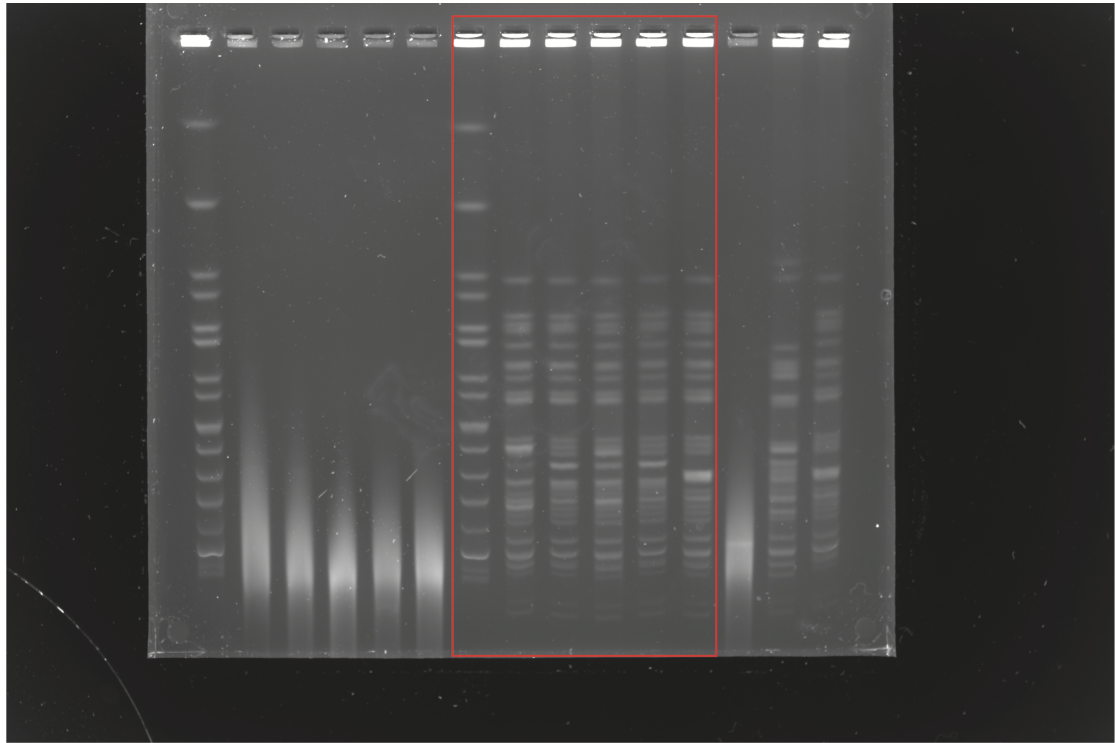

Supplement: Supplementary file 2 — Additional file 2: Figure S2. Original PFGE Pattern. [file 12941_2024_686_MOESM2_ESM.pdf]
