# Supplementary material for: Genomic insights into the evolution and mechanisms of carbapenem-resistant hypervirulent Klebsiella pneumoniae co-harboring blaKPC and blaNDM: implications for public health threat mitigation
Source: Ann Clin Microbiol Antimicrob. 2024 Mar 29;23:27. doi: 10.1186/s12941-024-00686-3 (PMC10981300; doi:10.1186/s12941-024-00686-3)
Supplement: Supplementary file 3 — Additional file 3: Table S1. The primers used in this study. [file 12941_2024_686_MOESM3_ESM.docx]

| **Table S1** The primers used in this study | | | |
| --- | --- | --- | --- |
| plasmid |  | primer name | 5'-3' sequence |
| pJ-JNKPN26-2_HNK |  | p2-U-F | atgaatgatgaagaactcgaacttg |
|  |  | p2-U-R | acaggcgaatataacgcat |
|  |  | p2-D-F | cccagcctcataggatt |
|  |  | p2-D-R | gaggttccagagcgacc |
| pJ-JNKPN26-3_HNK |  | p3-U-F | taaaaccacgacactggtgaagta |
|  |  | p3-U-R | acgccagaaaagatgcctc |
|  |  | p3-D-F | aatacacaggcgaatataacgcat |
|  |  | p3-D-R | ggatagccctgttgtatgccaa |
| pJ-JNKPN26-4_HNK |  | p4-U-F | aatggcctgggggtgtaa |
|  |  | p4-U-R | aacggacgctattatgaaccg |
|  |  | p4-D-F | gtggtccaggtaacggaag |
|  |  | p4-D-R | gcgcagcataacgattttttcatc |
|  |  | KPC-2-F | gtcgcggaaccattcg |
|  |  | KPC-2-R | agcaacaaattggcggc |
|  |  | NDM-1-F | gattgcgacttatgccaatgc |
|  |  | NDM-1-R | atgctggccttgggg |
